# Combining disciplines in the field of modern microbiology: a quantitative analysis of the literature on sporotrichosis

**DOI:** 10.3389/fcimb.2026.1742934

**Published:** 2026-04-20

**Authors:** Donglin Yuan, Shuang Bai, Yu Xing, Yuheng Cao, Bingbing Shang, Binbin Hou

**Affiliations:** 1 Department of Dermatology, The Second Hospital of Dalian Medical University, Dalian, Liaoning, China; 2 Department of Dermatology, The Affiliated Hospital of Inner Mongolia Medical University, Hohhot, China; 3 Emergency Department, The Second Hospital of Dalian Medical University, Dalian, Liaoning, China

**Keywords:** bibliometrics, data analytics, network analysis, quantitative analysis, *Sporothrix*, sporotrichosis

## Abstract

**Background:**

Sporotrichosis, a chronic infectious disease caused by the *Sporothrix schenckii* complex, has seen a rising incidence globally due to environmental changes and an increasing population of immunocompromised individuals, presenting a significant public health challenge.

**Objective:**

This study aims to elucidate the primary research themes, influential authors and institutions, and the evolution of sporotrichosis research trends through bibliometric analysis. In addition, we employ Latent Dirichlet Allocation (LDA) for topic modeling to identify potential research avenues and conceptual associations, while text mining analysis is used to uncover research gaps.

**Methods:**

A comprehensive literature search was conducted using PubMed, Scopus, and Web of Science Core Collection (1975–2024), yielding 1299 records. Various analytical tools, including Citespace, VOSviewer, and R’s bibliometrix package, were used for data analysis and visualization. The LDA algorithm was applied for topic modeling.

**Results:**

The analysis revealed an increasing trend in publications and citations, with Brazil leading in research output. Key authors and journals were identified, highlighting a shift in research focus from epidemiology and clinical manifestations to treatment methods, drug development, and pathogen classification. Topic modeling identified five major research directions.

**Conclusion:**

This bibliometric analysis highlights the interdisciplinary nature of sporotrichosis research and identifies critical areas for future investigation, including pathogenesis, diagnostic improvements, and novel therapeutic strategies. Enhanced international collaboration and resource sharing are essential to address the challenges posed by sporotrichosis, ultimately improving public health outcomes related to this disease.

## 1 Introduction

Sporotrichosis is a chronic infectious disease caused by the *Sporothrix schenckii* complex, which presents significant public health concerns globally (Rodrigues et al., 2022a). The prevalence of sporotrichosis varies markedly across geographical regions, with particularly high incidence rates observed in tropical and subtropical areas such as Brazil and Peru (Hernández-Castro et al., 2022). However, disease distribution is influenced by multiple interacting factors, including species-specific virulence, transmission mechanisms (e.g., zoonotic versus environmental exposure), host immune status, and antifungal susceptibility profiles, rather than geographic location alone. Here, the warm and humid climate creates an ideal environment for the survival and proliferation of *Sporothrix*, leading to increased infection rates (Dos Santos et al., 2024). Longstanding epidemic outbreaks of sporotrichosis have been reported in several Brazilian states since the late 1990s, particularly in Rio de Janeiro and Rio Grande do Sul, where sustained increases in case numbers have been observed over extended periods (Munhoz et al., 2022).

The rising incidence of sporotrichosis is exacerbated by global environmental changes, including elevated temperatures and altered humidity patterns, alongside a growing population of immunocompromised individuals, such as those living with HIV/AIDS, organ transplant recipients, and long-term users of immunosuppressive therapies (Alvarez et al., 2022; Duarte-Escalante et al., 2022). This demographic shift presents a significant challenge to public health, as sporotrichosis can affect multiple organ systems in humans.

Clinically, cutaneous and lymphocutaneous sporotrichosis represent the most prevalent clinical forms. These presentations typically involve localized lesions and may cause discomfort or functional limitations; severe systemic impact is primarily associated with disseminated or extracutaneous disease in immunocompromised individuals (Sharma et al., 2021; Zhu et al., 2023). If left untreated, the disease may progress to lymphocutaneous sporotrichosis, characterized by lymphangitis and lymphadenopathy (Schwalb et al., 2022). Ocular sporotrichosis, although rare, can result in irreversible vision damage, including blindness (Arinelli et al., 2023). Immunocompromised individuals are particularly susceptible to disseminated sporotrichosis, which can involve vital organs such as the lungs and bones, often resulting in severe outcomes and elevated mortality rates (Pinto-Almazán et al., 2023). Furthermore, domestic cats represent the principal zoonotic transmission source in hyperendemic regions, particularly in Brazil, where feline-associated transmission has played a central role in sustained outbreaks. In contrast, dogs appear to have a comparatively minor epidemiological role in human transmission (Dumont-Viollaz et al., 2025).

Although substantial progress has been made in elucidating host–pathogen interactions and immunological mechanisms in sporotrichosis (Albuquerque et al., 2021; Almeida-Paes et al., 2024; Rodrigues et al., 2022b), certain aspects of immune modulation and species-specific pathogenic variability remain areas of ongoing investigation. Current diagnostic methods exhibit limitations in sensitivity and specificity (de Carvalho et al., 2022), and the emergence of antifungal drug resistance complicates treatment strategies (Morgado et al., 2024). Therefore, it is essential to explore the underlying mechanisms of sporotrichosis, enhance diagnostic approaches, and develop more effective therapeutic options.

This study employs bibliometric analysis to investigate the primary themes, influential authors, and institutions in sporotrichosis research, as well as the evolution of research trends. By employing the Latent Dirichlet Allocation (LDA) model for topic modeling, we aim to uncover potential research themes and conceptual connections within the literature, thereby generating new insights into underexplored areas of sporotrichosis research. In addition, text mining analysis will identify overlooked topics, indicating promising directions for future investigation.

## 2 Materials and methods

### 2.1 Data download

**Figure 1:**
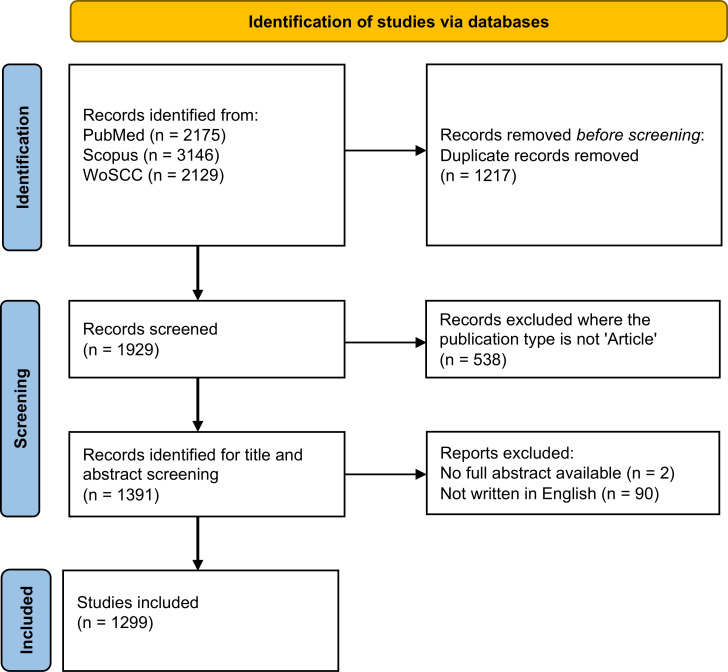
Search strategy and analysis flowchart for research in the field of sporotrichosis.

A thorough literature search was conducted using PubMed, Scopus, and Web of Science Core Collection (WoSCC), databases from January 01, 1975, to December 31, 2024. The primary search term employed was “sporotrichosis,” with a restriction to English-language publications and inclusion limited to original research articles. We additionally evaluated whether inclusion of the genus term “*Sporothrix*” would substantially expand the disease-relevant corpus. Preliminary screening indicated that many records retrieved using the genus term alone were related to environmental isolates, ecological studies, or general fungal biology without direct clinical or epidemiological focus. Given that the objective of this study was to analyze disease-centered research trends, the search strategy was intentionally restricted to “sporotrichosis” to ensure thematic coherence and to avoid dilution of the dataset with non-disease-oriented publications. To mitigate the impact of database updates, all data results were downloaded on the same day, and the formats included plain text, BibTex, and Tab delimited files. Ultimately, a total of 1299 records were identified for the bibliometric analysis in this study. The specifics of the retrieval and filtering processes are illustrated in **Figure 1**.

### 2.2 Data analysis and visualization

In this study, we used various analytical tools, including Citespace (version 6.3.R1), VOSviewer (version 1.6.20), the bibliometrix package (version 4.3.0) in R (version 4.4.0), the KH Coder software (version 3b07d), and the online platform for scientific bibliometrics (https://bibliometric.com/) to examine published studies and generate visualization maps (Tomaszewski, 2023).

VOSviewer is capable of visualizing knowledge graphs, encompassing co-authorship analysis, co-occurrence analysis and co-citation analysis across extensive literature datasets, thereby representing authors, journals and other pertinent information. This tool has been extensively employed in bibliometric analysis research (Van Eck and Waltman, 2010). In these visualizations, distinct nodes signify authors, countries, organizations, journals and keywords, with the size of each node reflecting the frequency of citations. The connections between nodes illustrate collaborative and co-occurring relationships, while the colors of the nodes and lines denote different clusters or corresponding years or average references. In addition, we conducted a reference clustering analysis using CiteSpace (version 6.3.R1) to discern advancements and prospective trends within the research domain (Du et al., 2024).

Furthermore, we established a network illustrating international collaborations among countries through the online scientometrics platform. The bibliometrix package in R facilitates the creation of historiograph plots of references, which elucidate citation relationships among the literature, thereby revealing the mechanisms of knowledge transfer within the research field (Arruda et al., 2022). By analyzing citation chains, we can trace the dissemination of significant theories or discoveries. Each node in this network represents a piece of literature, with connecting lines indicating citation relationships. Arrows directed towards the cited literature signify the flow of knowledge from the citing literature to the cited literature, while the timeline in the graph typically illustrates the chronological progression from left to right.

Topic modeling, a technique within Natural Language Processing (NLP), is employed to uncover potential topics within open literature. Latent Dirichlet Allocation (LDA) is a widely used method for topic modeling that effectively manages substantial volumes of unstructured text (Jelodar et al., 2019). LDA functions by generating a vocabulary of term functions and analyzing the co-occurrence of word frequencies within documents. Following the construction of the vocabulary, LDA assesses the relevance of an article to a specific topic based on the word frequencies present in each document. The determination of the number of topics is based on various criteria, including the paired cosine distance method, the Kullback-Leibler divergence method, and model coherence. Specific results are illustrated in **Supplementary Figure 1**, with further details provided in the **Supplementary Material**.

The raw data obtained from the Web of Science Core Collection were initially imported into Microsoft Excel 2021 for preliminary organization. We selected only the titles and abstracts of each article to form the original corpus. To enhance the reliability and validity of the findings, we implemented word frequency thresholds, eliminated generic stop words, and specified particular stop words. LDA topic modeling and word cloud visualization were conducted using KH Coder software and WordCloud. In the final phase, we manually assigned names to each topic based on the ten most significant articles and twenty key terms associated with each topic.

## 3 Results and discussion

### 3.1 Annual trends in publications

**Figure 2:**
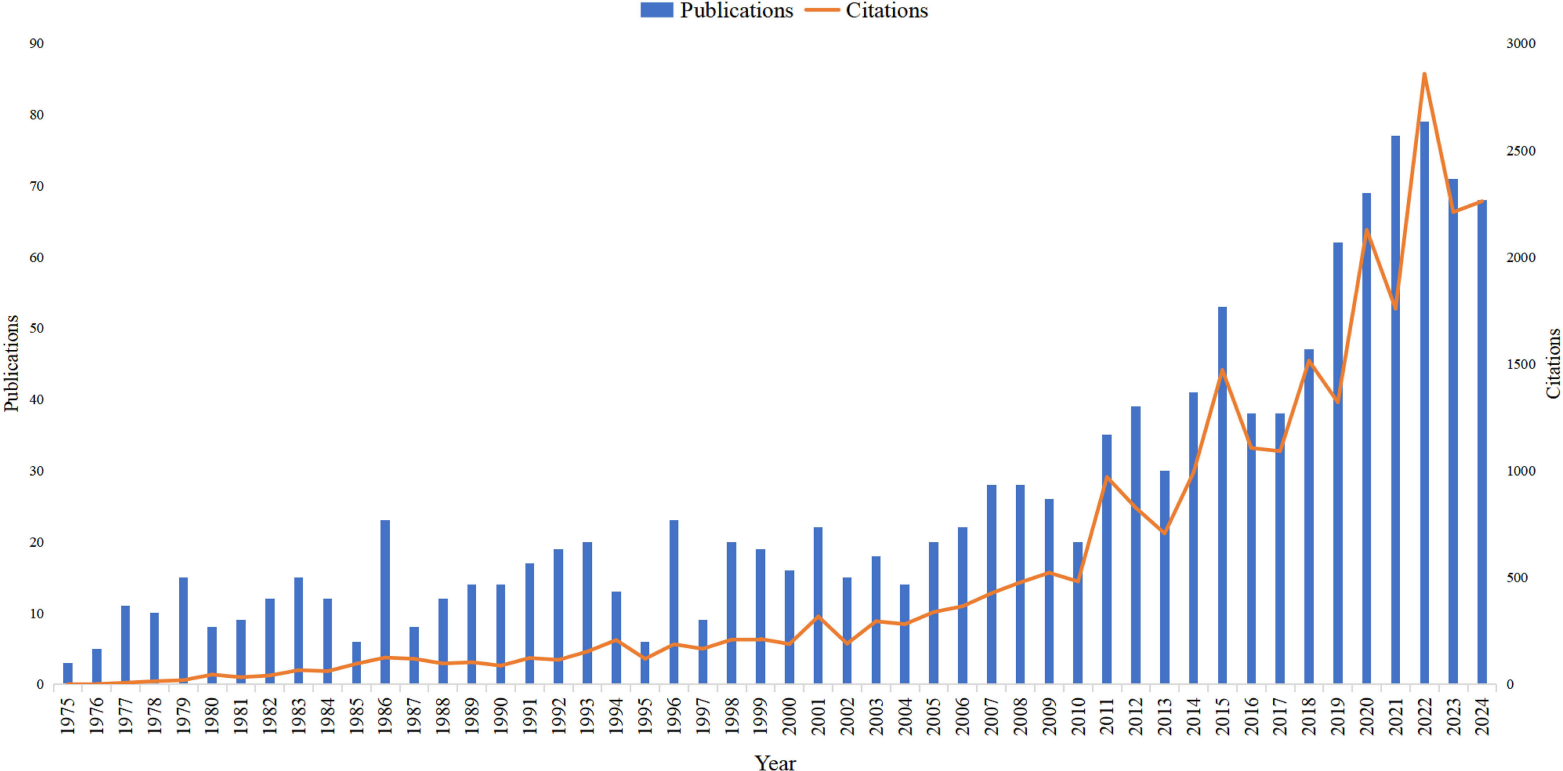
Annual growth in publications and citations in sporotrichosis research.

An analysis of the annual number of publications and citations in the field of sporotrichosis from 1975 to 2024 was conducted to elucidate trends and the significance of this research area (**Figure 2**).

From 1977 to 1990, the overall volume of publications was low and exhibited minimal fluctuation. Correspondingly, the number of citations was also relatively modest, peaking at 50 in 1988, which suggests that sporotrichosis did not garner substantial attention during this timeframe.

In the subsequent period from 1991 to 2005, there was a gradual increase in the number of publications, rising from approximately 100 in 1991 to around 200 in 2005. Although citations also experienced an uptick, the growth was modest, likely reflecting the nascent stage of research interest in this domain.

Between 2006 and 2015, both the number of publications and citations demonstrated a more pronounced increase. Publications surged from 250 in 2006 to approximately 400 by 2015, while citations escalated from several hundred to thousands. This growth can be attributed to several factors: advancements in medical technology that have opened new avenues for research on the diagnosis and treatment of sporotrichosis, as well as a heightened global awareness of fungal infectious diseases, which has encouraged more researchers to engage in sporotrichosis studies.

From 2016 to 2024, publications increased from 38 to 68, peaking at 79 in 2021. Citations grew dramatically from 1,091 to 2,261, reaching a high of 2,857 in 2021. This indicates expanding research activity and growing academic impact in sporotrichosis studies, with peak momentum around 2019-2021.

Overall, the increasing trend in the number of publications over the past decades suggests a growing interest among researchers in sporotrichosis. This rise can be attributed to several factors: an increase in the incidence of sporotrichosis, which has drawn the attention of the medical community; advancements in biotechnology that have provided enhanced methodologies for studying sporotrichosis; and the strengthening of international collaboration and exchange, which has facilitated research progress in this area.

The observed increase in citations is directly correlated with the rise in the number of publications, suggesting that research outcomes in this domain have garnered increasing attention and recognition. The elevated volume of citations further indicates that the research conducted in this area possesses significant academic merit and clinical relevance, thereby serving as a valuable reference for future investigations. Variations in the quantity of publications and citations may be attributed to a multitude of factors, including shifts in research priorities, the influence of significant events, and advancements in research methodologies. For example, a decrease in publication numbers during certain years may be attributed to a diversion of interest towards other related fields or a stabilization phase within the current research area.

In summary, the trends in publication and citation counts related to sporotrichosis research from 1977 to 2024 show significant fluctuations. The overall upward trajectory in publication numbers signifies an increasing emphasis on this research area, while the concurrent rise in citations underscores the substantial academic and clinical importance of the findings. Looking ahead, as awareness of fungal infectious diseases continues to grow and research methodologies evolve, it is anticipated that sporotrichosis research will further advance, thereby enhancing support for clinical diagnosis and treatment.

**Figure 3:**
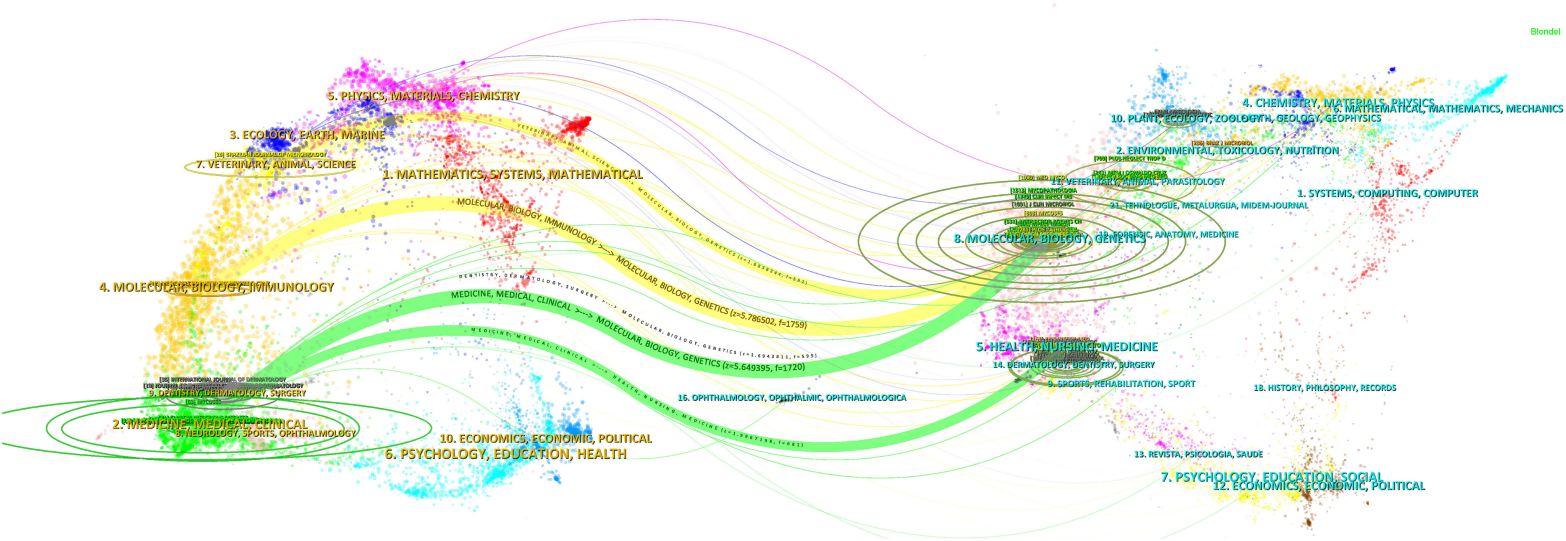
Disciplinary analysis double map overlay in sporotrichosis research.

The examination of the distribution of academic disciplines through the CiteSpace dual map overlay reveals that research pertaining to sporotrichosis is inherently interdisciplinary (**Figure 3**). Notably, the fields of “MATHEMATICS & SYSTEMS MATHEMATICS” and “COMPUTING, COMPUTER SYSTEMS” imply the application of mathematical modeling and computational techniques in data analysis and simulation, which may enhance the understanding of the pathogenesis and transmission dynamics of sporotrichosis. The emergence of the discipline “MOLECULAR IMMUNOLOGY” underscores a growing interest in investigating the immune mechanisms associated with sporotrichosis. Research in molecular immunology is poised to elucidate the host’s immune response to sporotrichosis, thereby providing a theoretical foundation for the development of novel therapeutic interventions. Furthermore, the domain of “HEALTH, NURSING, MEDICINE & TECHNOLOGY, DENTISTRY, SURGERY” encompasses a broad spectrum of medical disciplines, indicating that the study of sporotrichosis extends beyond mere diagnosis and treatment to include nursing and surgical considerations. The identification of the field “OPHTHALMOLOGY, OPHTHALMIC” suggests that ocular sporotrichosis, although infrequent, is gaining attention due to its potential to adversely affect vision, warranting further investigation. Additionally, the inclusion of the field “ECONOMICS, ECONOMIC, POLITICAL SCIENCE, PSYCHOLOGY, EDUCATION, HEALTH” indicates that the exploration of sporotrichosis transcends the confines of medicine, engaging with social sciences such as economics, psychology, and education. For example, an economic perspective may facilitate the analysis of healthcare resource consumption and the economic implications of sporotrichosis (Gonçalves et al., 2024), while psychological inquiries could address the mental health of affected individuals and their coping mechanisms (Mahajan, 2014). Although not a mental illness, disseminated sporotrichosis, especially when it affects the central nervous system, can cause neurological and psychiatric symptoms (Kauffman, 2019). It’s important to emphasize that most cases of sporotrichosis do not present with neurological or psychiatric symptoms. The symptoms mentioned above are more common in cases of disseminated sporotrichosis and usually occur in conjunction with other symptoms of infection, such as fever, weight loss, and fatigue.

In summary, research on sporotrichosis is characterized by a multidisciplinary approach, with collaborative efforts from diverse academic fields contributing to a more comprehensive understanding of the disease. Moving forward, it is imperative to promote interdisciplinary collaboration and communication to advance sporotrichosis research and to devise more effective strategies for its prevention and treatment.

### 3.2 Country and institutional analysis

#### 3.2.1 Country analysis

**Figure 4:**
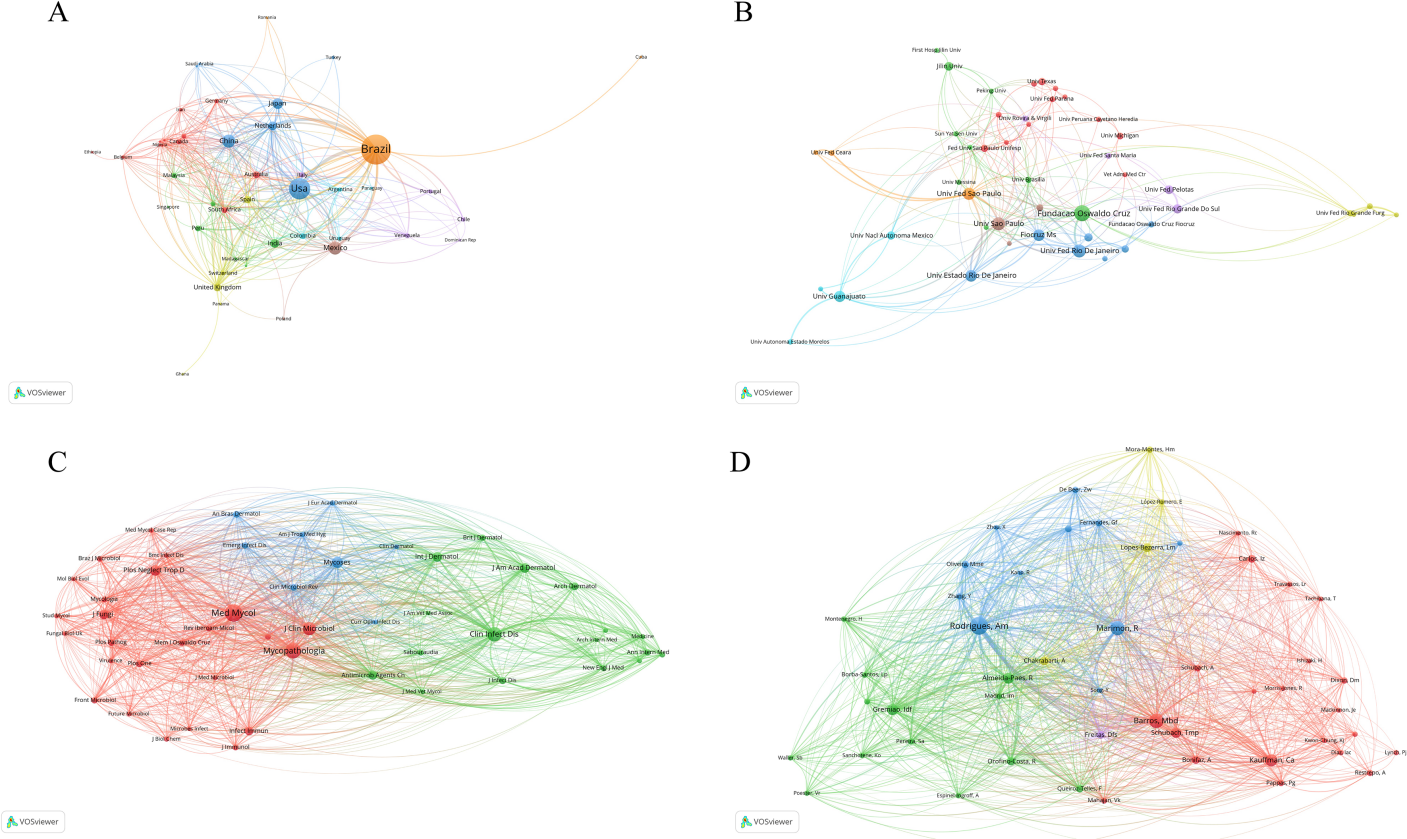
Comprehensive network analysis of co-authorship and co-citation in sporotrichosis research: national co-authorship **(A)**, institutional co-authorship **(B)**, author co-citation **(C)**, journal co-citation **(D)**.

**Figure 4A** illustrates notable disparities in the volume of publications pertaining to sporotrichosis research across various nations. Brazil leads with a total of 529 publications, underscoring its significant engagement and prominence in this area of study. Brazil’s disproportionately high research output is closely associated with the large-scale zoonotic outbreaks of *Sporothrix brasiliensis* reported over the past two decades, particularly in Rio de Janeiro and other southeastern states. The hyperendemic pattern of feline-associated transmission has generated sustained public health concern, thereby stimulating continuous clinical, epidemiological, and molecular investigations. In addition, Brazil hosts internationally recognized research institutions such as Fundação Oswaldo Cruz (Fiocruz) and the Federal University of Rio de Janeiro (UFRJ), both of which have established strong traditions in medical mycology and fungal epidemiology. These epidemiological and institutional factors likely contribute to Brazil’s leading position in global sporotrichosis research. It should be noted that epidemiological publications prior to the widespread implementation of molecular taxonomy (approximately before 2006) often relied on phenotypic identification methods. Therefore, earlier species-level classifications in the literature should be interpreted with caution when comparing historical and contemporary datasets. The United States follows with 273 publications, indicating its substantial role in sporotrichosis research as well. Other countries, including the Netherlands, India, the United Kingdom, Spain, and Japan, also contribute meaningfully to the literature, with publication counts of 48, 47, 36, 32, and 69, respectively. These nations possess robust capabilities and resources in medical research, thereby facilitating their contributions to the understanding of sporotrichosis. On the other hand, some countries (such as Austria, Belgium, Egypt, Ethiopia, Ghana, Nigeria, Romania, Sudan, and the United Arab Emirates) exhibit a lower publication output, with only 2 publications each. This discrepancy may be attributed to a relative scarcity of medical research resources, investment, and focus on sporotrichosis within these nations. Overall, the distribution of sporotrichosis-related publications demonstrates variability across countries. While higher publication volumes are observed in certain nations, bibliometric analysis reflects research output within indexed databases and does not directly measure public health impact, infrastructure capacity, funding levels, or scientific contribution. Notably, several countries traditionally classified as developing nations-including Brazil, China, India, and Peru—have made substantial contributions to the literature, particularly in areas such as epidemiology, molecular characterization, and clinical management. Therefore, publication volume should be interpreted as an indicator of indexed research activity rather than a proxy for national scientific capacity or public health engagement.

In conclusion, an analysis of the publication distribution in sporotrichosis research reveals a global imbalance. Future efforts should prioritize enhancing international collaboration and exchange to facilitate resource sharing and technical cooperation, thereby elevating the global research capacity and efficacy in the prevention and control of sporotrichosis. In addition, it is imperative for developing countries to augment their investments in medical research and bolster their scientific research capabilities to contribute more significantly to global public health initiatives.

#### 3.2.2 Institutional analysis

**Figure 4B** illustrates a significant disparity in the volume of publications across various research institutions within the field of sporotrichosis. An econometric analysis of the literature pertaining to sporotrichosis has revealed several institutions that have made notable contributions to this area of study. Notably, Fiocruz in Brazil leads with 50 publications, reflecting a substantial commitment to sporotrichosis research and yielding considerable outcomes. Additionally, the Federal University of Rio de Janeiro and the University of São Paulo, both also located in Brazil, demonstrate robust research capabilities with 61 publications each. Other institutions contributing to sporotrichosis research include the National Autonomous University of Mexico with 24 publications, the Peruvian University Cayetano Heredia with 11 publications, and the University of Michigan in the United States with 18 publications, all of which have played significant roles in advancing knowledge in this field. Geographically, institutions engaged in sporotrichosis research are distributed across multiple countries, including the United States, Brazil, Mexico, Peru, and China, indicating that sporotrichosis is a subject of global interest, with research institutions worldwide actively allocating resources to this area. Among the institutions with a high publication output, several Brazilian universities and research entities are prominent, such as the Federal University of São Paulo with 55 publications and the Federal University of Ceará with 13 publications. This trend may be attributed to Brazil’s geographical and climatic conditions, which render sporotrichosis relatively prevalent in the region, thereby motivating local research institutions to focus more intently on this disease. Moreover, some Chinese institutions have also made contributions to sporotrichosis research, including Jilin University with 27 publications, the First Hospital of Jilin University with 9 publications, and Sun Yat-sen University with 11 publications, indicating that China holds a significant position within the global sporotrichosis research landscape.

As such, the bibliometric analysis of research institutions in the field of sporotrichosis reveals a relatively dispersed research capacity, although some institutions exhibit exceptional contributions. Moving forward, it is imperative to enhance collaboration and communication among research institutions across different countries to collectively advance the comprehensive development of sporotrichosis research.

### 3.3 Authors’ analysis

**Figure 4C** illustrates a significant variation in citation counts among authors, with Zoilo Pires De Camargo emerging prominently with 1,930 citations, indicative of the widespread recognition of his contributions to sporotrichosis research. Similarly, Anderson Messias Rodrigues, with 2,407 citations, underscores his significance in the field. In addition, authors such as Thais Maria Pereira Schubach, with 895 citations, and Rosely Maria Zancope-Oliveira, with 1,182 citations, have also made substantial contributions to the study of sporotrichosis. The authorship landscape encompasses researchers from various countries, highlighting the international dimension of sporotrichosis research. Notably, contributors like Rodrigo Almeida-Paes, Fernando Almeida-Silva, and Mario Carlos Araujo Meireles, represent diverse national backgrounds and have enriched the field. The research outputs of these highly cited authors often delineate the forefront and emerging trends within the discipline. A comprehensive examination of their studies can yield valuable insights into the latest advancements in the pathogenesis, diagnostic methodologies, and therapeutic approaches to sporotrichosis. In conclusion, the bibliometric analysis of prominent authors in sporotrichosis reveals a cohort of distinguished researchers who are actively advancing the field. Future efforts should focus on enhancing support and collaboration with these key research figures to facilitate the continued progression of sporotrichosis research.

### 3.4 Journal analysis

**Figure 4D** illustrates a significant disparity in citation counts among various journals, with Medical Mycology emerging as the most cited journal, including a total of 1983 citations (**Table 1**). This indicates the journal’s pivotal role in the academic dissemination of research pertaining to sporotrichosis. Other highly cited journals include Mycopathologia, with 1651 citations; the Journal of Clinical Microbiology, which has 1151 citations; Mycoses, with 925 citations; and the Journal of the American Academy of Dermatology, which has 552 citations. In addition, specialized dermatology journals such as Anais Brasileiros de Dermatologia, British Journal of Dermatology, and International Journal of Dermatology contribute significantly to the dissemination of sporotrichosis research, with citation counts of 304, 230, and 54, respectively. Journals focused on infectious diseases, including Antimicrobial Agents and Chemotherapy and Emerging Infectious Diseases, have also garnered attention, with citation totals of 399 and 157, respectively. The disciplinary distribution of these journals encompasses a range of fields, including tropical medicine, dermatology, and microbiology, highlighting the multidisciplinary nature of sporotrichosis research. Notably, tropical medicine journals such as the American Journal of Tropical Medicine and Hygiene, Memórias do Instituto Oswaldo Cruz, and Revista da Sociedade Brasileira de Medicina Tropical serve as essential academic platforms for the study of sporotrichosis in tropical regions. Highly cited journals typically represent essential academic resources and emerging research trends within the field. Analyzing these journals facilitates a deeper understanding of the academic dynamics and developmental trajectories of sporotrichosis research. Furthermore, this analysis provides valuable insights for researchers regarding journal selection for manuscript submission and access to the latest research findings. In summary, the bibliometric analysis of journals publishing sporotrichosis research reveals a concentration of findings within a select group of influential professional journals. It is imperative to further enhance the leading role of these journals in sporotrichosis research to foster academic communication and advancement in this domain.

**Table 1:** Impact and contribution metrics of leading countries, authors, institutions, and journals in sporotrichosis research.

Ranking		NC	NP	AC
Journals				
1	Medical Mycology	1983	82	24.1829
2	Mycopathologia	1651	86	19.1977
3	Clinical Infectious Diseases	1498	21	71.3333
4	Journal of Clinical Microbiology	1151	16	71.9375
5	Plos Neglected Tropical Diseases	985	27	36.4815
6	Mycoses	925	63	14.6825
7	Journal of the American Academy of Dermatology	552	19	29.0526
8	International Journal of Dermatology	546	35	15.6
9	Antimicrobial Agents and Chemotherapy	399	11	36.2727
10	Infection and Immunity	386	7	55.1429
Country				
1	Brazil	12099	529	43.0723
2	USA	7277	273	49.2
3	Netherlands	2107	48	51.6923
4	Mexico	1908	100	134.2
5	Spain	1801	32	16.45
6	Japan	1547	69	22.8333
7	China	1474	102	12.4828
8	Peru	958	20	38.6667
9	United Kingdom	870	36	37.1429
10	South Africa	766	22	27.375
Institutions				
1	Univ Fed Sao Paulo	2259	55	41.0727
2	Fundacao Oswaldo Cruz	2105	92	22.8804
3	Fiocruz	1953	50	39.06
4	Univ Estado Rio De Janeiro	1682	48	35.0417
5	Univ Fed Rio De Janeiro	1568	61	25.7049
6	Univ Rovira & Virgili	1386	14	99
7	Univ Sao Paulo	1287	61	21.0984
8	Univ Michigan	1070	18	59.4444
9	Univ Texas	895	21	42.619
10	Cbs Knaw Fungal Biodivers Ctr	876	10	87.6
Authors				
1	Rodrigues, Anderson Messias	2407	49	49.1224
2	De Camargo, Zoilo Pires	1930	38	50.7895
3	Almeida-Paes, Rodrigo	1246	64	19.4688
4	Zancope-Oliveira, Rosely Maria	1182	52	22.7308
5	Schubach, Thais Maria Pereira	895	13	68.8462
6	Gutierrez-Galhardo, Maria Clara	821	33	24.8788
7	Pereira, Sandro Antonio	813	27	30.1111
8	De Hoog, G. Sybren	792	12	66
9	Wanke, B	750	10	75
10	Fernandes, Geisa Ferreira	740	10	74

NP, number of publications; NC, number of citations; AC, average citations(NC/NP).

### 3.5 Keyword analysis

**Figure 5:**
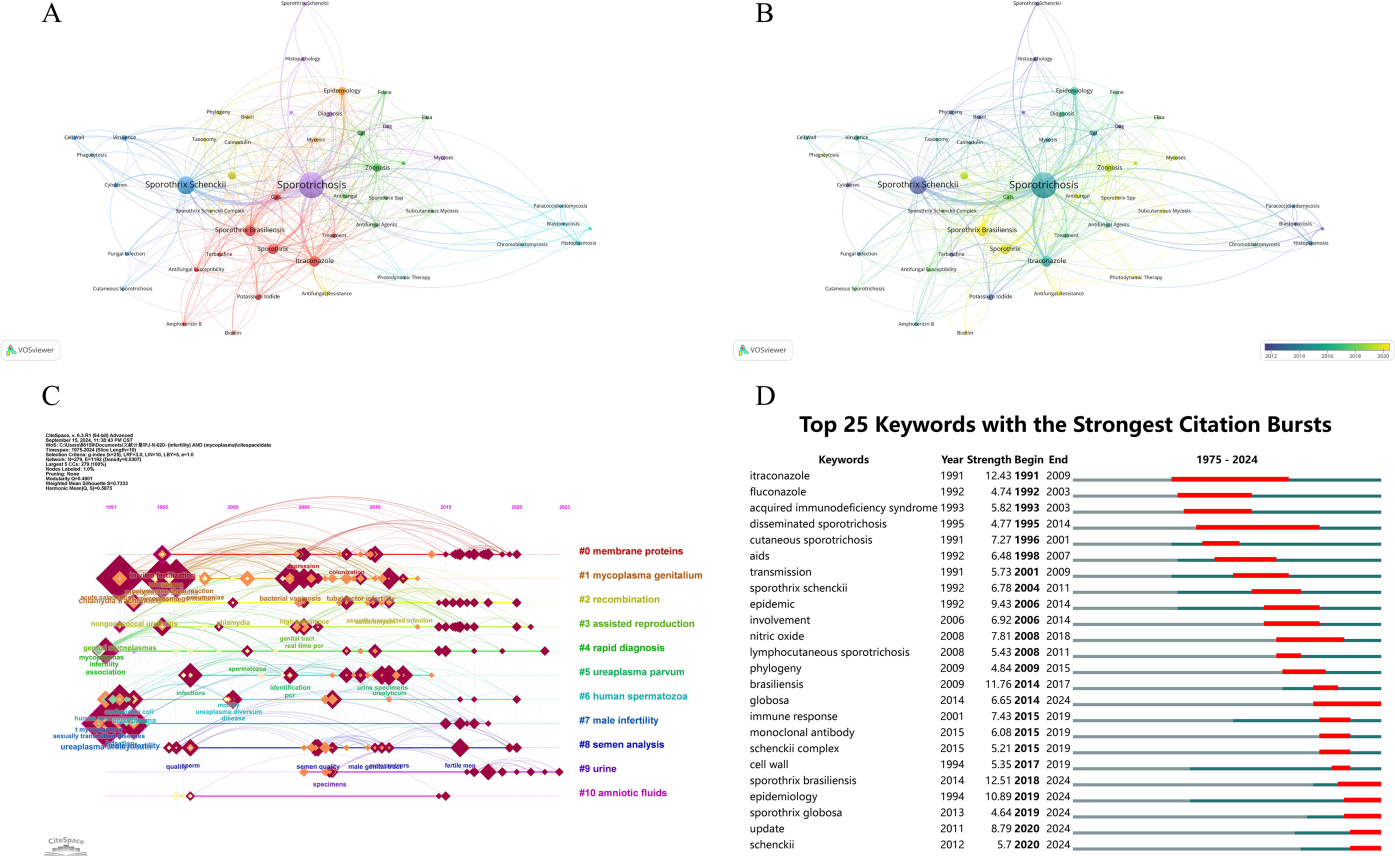
Clustering grid analysis of keyword co-occurrence based on VOSviewer **(A)**, temporal dimension grid analysis of keyword co-occurrence based on VOSviewer **(B)**, Citespace keyword clustering timeline view **(C)**, and citation explosion (top 25 keywords) **(D)**.

**Figure 5** presents the results of the co-occurrence network (A) and temporal overlay (B) analysis of keywords in the literature in the field of sporotrichosis relationships using VOSviewer software. These graphs reveal the links between the main research themes in the field and their temporal evolution characteristics.

In the clustered network analysis (**Figure 5A**), the size and color of the nodes represent the frequency of occurrence of the keywords and the strength of the relationship between the research themes. The analysis reveals several significant themes, including “*Sporothrix schenckii*,” “sporotrichosis,” “treatment,” “diagnosis,” and “epidemiology.” The prominence of these themes is indicated by the larger nodes, which signify a high frequency of occurrence within the study. The multiple connections between these nodes suggest a strong interrelationship among the research themes. **Figure 5A** further illustrates the clustering of these research themes. Notably, “*Sporothrix schenckii*” and “sporotrichosis” exhibit a close association, underscoring the research emphasis on both the pathogen and the associated disease. Additionally, the term “treatment” is intricately linked to “drugs” and “surgery,” indicating a range of therapeutic approaches for sporotrichosis. Furthermore, the term “diagnosis” is associated with “laboratory tests” and “clinical manifestations,” highlighting the comprehensive nature of diagnostic methodologies employed in this field.

In the temporal dimension network analysis (**Figure 5B**), the colors of the nodes reflect the research activity of the keywords at different times. Initial investigations (represented in blue and green) primarily concentrated on subjects such as “epidemiology” and “clinical manifestations,” indicating that the principal aim of research during that period was to elucidate the epidemiological aspects and clinical presentations of filariasis. As time progressed, the focus of research has gradually transitioned towards more contemporary themes, such as “treatment” and “new drugs,” denoted in red and yellow, which have emerged as prominent areas of inquiry.

Using keyword co-occurrence analysis through VOSviewer, as illustrated in **Figures 5A, B**, one can distinctly observe the evolution of research within the domain of sporotrichosis. The initial emphasis on the epidemiology and clinical manifestations of the disease has progressively shifted towards the investigation of therapeutic strategies and the innovation of new pharmacological agents. This trend signifies a deepening comprehension of sporotrichosis among researchers and their dedication to developing more effective treatment modalities for affected individuals. Looking ahead, as research endeavors persist, we anticipate significant advancements in the diagnosis, treatment, and prevention of sporotrichosis.

The redder color of the cluster labels (**Figure 5C**) indicates that the research is closer to the present. For example, labels #4, #5, #7, and #8 exhibit a pronounced red hue, signifying that these topics have garnered increased attention in recent research pertaining to sporotrichosis. Label #0 likely pertains to investigations of cell wall-associated proteins (e.g., GP70) and related surface antigens in *Sporothrix*, which formed an important foundation in earlier immunological studies but have received comparatively less recent emphasis. Labels #7 and #8 relate to terms such as male infertility and semen analysis. Although these clusters may appear disconnected from core infection biology, their presence in the dataset most likely reflects multidisciplinary publication environments and journal indexing practices rather than established pathogenetic or clinical associations with sporotrichosis. Given the bibliometric nature of this study, clustering results are generated from probabilistic keyword co-occurrence patterns across titles and abstracts; therefore, thematic proximity does not imply etiological linkage or diagnostic relevance. These peripheral clusters should be interpreted cautiously as potential methodological artifacts rather than evidence of a clearly defined emerging clinical trajectory. Further targeted investigation would be necessary to determine whether such signals represent genuine thematic expansion or simply statistical noise within large-scale text mining processes. The diversity and evolving nature of sporotrichosis research are clearly illustrated through the cluster analysis conducted by CiteSpace. The various clustering labels signify distinct research trajectories and focal points, indicating a gradual shift in research emphasis towards rapid diagnosis, pathogen identification, and studies related to the reproductive system over time. These findings offer significant insights and guidance for future in-depth investigations into sporotrichosis.

**Figure 5D** illustrates the top 25 keywords exhibiting the most significant citation bursts from 1975 to 2024. Notably, “itraconazole” experienced a pronounced citation burst in 1991, with an intensity of 12.43, persisting until 2009. This surge reflects intensified scholarly discussion regarding itraconazole as the primary antifungal therapy for sporotrichosis during that period. In addition, citation bursts for “fluconazole” and “aids” emerged in 1992. Fluconazole began to be investigated as an alternative antifungal agent for its efficacy in treating sporotrichosis, while the emergence of “aids” signified a growing interest in sporotrichosis among immunocompromised individuals. In 1993, “acquired immunodeficiency syndrome” exhibited a citation burst intensity of 5.82, lasting until 2003, thereby underscoring the connection between immunodeficiency and sporotrichosis. A citation burst of 4.77 for “disseminated sporotrichosis” in 1995, which continued until 2014, reflects an increased scholarly focus on severe manifestations of sporotrichosis. Furthermore, “cutaneous sporotrichosis” recorded a high citation burst intensity of 7.27 in 1991, persisting until 2001, indicating that this form of sporotrichosis has been a primary research focus.

The citation surge for “epidemic” commenced in 1992 with an intensity of 9.43 and extended until 2014, suggesting that epidemiological investigations of sporotrichosis have been significant at various intervals. Citation bursts for “involvement” and “nitric oxide” in 2006 highlight research concerning the extent of sporotrichosis involvement and the role of nitric oxide in its pathogenesis, respectively. The terms “lymphocutaneous sporotrichosis” and “phylogeny” also gained traction, indicating that the study of specific sporotrichosis variants and the phylogenetic analysis of the pathogen became prominent topics during this period. Following 2009, keywords such as “brasiliensis” and “globosa” experienced a surge in citations, reflecting the increasing importance of research on different *Sporothrix* species within the clinical clade. In 2015, there was a notable increase in citations for “monoclonal antibody” and “schenckii complex,” underscoring the significance of novel therapeutic strategies and comprehensive research on the classification of pathogenic organisms. After 2019, “epidemiology” re-emerged as a focal point with an intensity of 10.89, lasting until 2024, indicating that epidemiological research on sporotrichosis remains a critical area of inquiry. In summary, an analysis of the citation bursts related to sporotrichosis reveals the evolution of research hotspots over time. The initial emphasis on therapeutic agents and immunocompromised populations has transitioned to a focus on various sporotrichosis forms, the classification of pathogenic organisms, and innovative therapeutic approaches, culminating in a sustained interest in epidemiology in recent years. This trajectory reflects the ongoing advancement and deepening of research in the field of sporotrichosis. Future investigations are anticipated to yield significant breakthroughs in the diagnosis, treatment, and prevention of this condition.

Furthermore, an examination of the frequency of keywords and their corresponding total link strength values (**Table 2**) reveals that the term “Sporotrichosis” occupies the foremost position, with 438 occurrences and a total link strength of 640. This finding underscores the significance of sporotrichosis as a primary focus of research within this domain. The keyword “*Sporothrix schenckii*” follows, with 206 occurrences and a total link strength of 252, highlighting the critical role of this pathogen in sporotrichosis studies. The term “Itraconazole” appears 78 times, with a total link strength of 164, indicating substantial interest in this antifungal agent as a treatment for sporotrichosis. Additionally, the occurrences of “*Sporothrix brasiliensis*” and “*Sporothrix globosa*” suggest an increasing recognition of the relevance of various strains in sporotrichosis research. The keyword “Zoonosis” was noted 49 times, with a total association strength of 104, implying the potential for zoonotic transmission of sporotrichosis, warranting further investigation. The term “Epidemiology” was recorded 44 times, with a total association strength of 97, reflecting concerns regarding the prevalence of sporotrichosis. The keywords “Cats” and “Cat” appeared 31 and 22 times, respectively, with total association strengths of 72 and 57, suggesting a possible role of felines in the transmission or infection of sporotrichosis. The term “Potassium Iodide” was mentioned 26 times, with a total association strength of 56, indicating that its application in the treatment of sporotrichosis has garnered some attention. The keyword “Treatment” was noted 17 times, with a total association strength of 40, signifying that research into the treatment of sporotrichosis is a critical area of inquiry. The occurrences of “Antifungal Resistance” and “Antifungal Susceptibility” reflect growing concerns regarding the drug resistance of pathogenic fungi. The significance of accurate diagnosis in sporotrichosis is underscored by the 20 occurrences of “Diagnosis,” which has a total link strength of 32. The term “Virulence” also appeared 20 times, with a total association strength of 32, suggesting that investigations into the virulence of pathogenic fungi may provide valuable insights into the disease’s pathogenesis. Other keywords, such as “Blastomycosis,” “Mycoses,” and “Phylogeny,” also indicate the interconnectedness of sporotrichosis with other fungal diseases and the phylogenetic studies of pathogenic organisms. In conclusion, the analysis of keywords within the field of sporotrichosis illustrates a diverse array of research focuses, encompassing the disease itself, the associated pathogens, treatment strategies, diagnostic approaches, epidemiological concerns, and relationships with other diseases.

**Table 2:** Frequency of keyword occurrence and total contact strength values.

Rank	Keyword	Occurrences	Total link strength	Rank	Keyword	Occurrences	Total link strength
1	Sporotrichosis	438	640	21	Virulence	20	32
2	Sporothrix Schenckii	206	252	22	Brazil	12	31
3	Itraconazole	78	164	23	Antifungal	15	30
4	Sporothrix Brasiliensis	91	164	24	Blastomycosis	9	30
5	Sporothrix	73	117	25	Mycoses	16	30
6	Zoonosis	49	104	26	Calmodulin	10	29
7	Epidemiology	44	97	27	Phylogeny	11	29
8	Cats	31	72	28	Feline	10	28
9	Sporothrix Globosa	43	59	29	Cytokines	18	26
10	Cat	22	57	30	Dog	11	25
11	Potassium Iodide	26	56	31	Antifungal Agents	11	24
12	Mycosis	17	45	32	Histopathology	14	24
13	Histoplasmosis	18	44	33	Taxonomy	10	24
14	Treatment	17	40	34	Amphotericin B	11	23
15	Antifungal Resistance	14	35	35	Sporothrix Schenckii Complex	10	22
16	Antifungal Susceptibility	16	35	36	Sporothrix Spp.	12	18
17	Coccidioidomycosis	9	33	37	Subcutaneous Mycosis	9	18
18	Terbinafine	15	33	38	Cell Wall	13	17
19	Diagnosis	20	32	39	Fungal Infection	12	17
20	Paracoccidioidomycosis	10	32	40	Sporothrix-Schenckii	15	17

**Figure 6:**
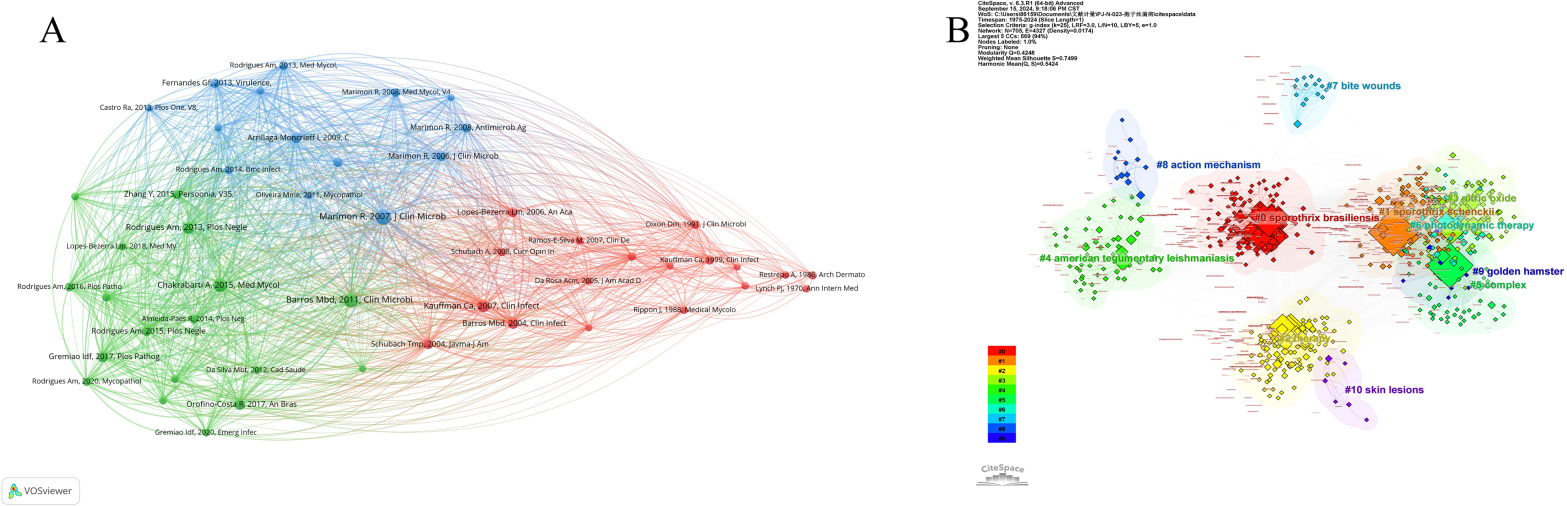
Vosviewer-based citation analysis **(A)** and citespace-based clustering view of co-cited reference files **(B)**.

The citation analysis conducted using VOSviewer (**Figure 6A**) has elucidated significant literature and author networks within the domain of sporotrichosis research. Notably, the works of Rodrigues A.M, published in Med Mycol in 2013, along with various studies disseminated across different journals in subsequent years, have been cited extensively, underscoring their pivotal role in advancing the understanding of sporotrichosis. Similarly, the contributions of Fernandes G.F., published in Virulence in 2013, and Marimon R., whose works appeared in journals such as Med Mycol and Antimicrob Agents in various years, also exhibited high citation frequencies. These seminal publications encompass a broad spectrum of topics related to sporotrichosis, including pathogen characterization, pathogenesis, diagnostic methodologies, and therapeutic approaches. For example, Marimon R’s 2008 article is recognized for its significant impact on the exploration of the pathogenic bacteria associated with sporotrichosis, while Rodrigues A.M’s 2013 research published in PLOS One may have offered novel insights into specific facets of the disease. A closer examination of the literature reveals that sporotrichosis research is characterized by a multifaceted approach, with various authors investigating the condition from diverse perspectives, thereby enriching the field with a comprehensive body of knowledge. Some studies concentrate on the clinical manifestations and diagnostic techniques pertinent to sporotrichosis, thereby establishing a foundation for accurate disease identification and diagnosis. Others emphasize the biological properties and pathogenesis of causative agents, contributing to a more profound comprehension of the disease’s progression. Furthermore, the citation analysis highlights the collaborative research network within this field. The interconnections among different authors and research institutions, as evidenced by citation patterns, reflect their cooperative efforts and communication in sporotrichosis research. Such collaboration facilitates the integration of resources across various stakeholders, thereby collectively advancing the progress of sporotrichosis research.

In the clustering view (**Figure 6B**) the different colors represent the various major research clusters, and the cluster labels in this study indicate that the research areas cover a wide range of topics, with the “bite wounds” (#7) cluster possibly indicating the direction of research on the association between *sporothrix* mycosis and bite wounds; the “ action mechanism” (#8) clustering may involve research on the mechanism of action of sporothrixomycosis; “*sporothrix* brasiliensis” (#1) and “sporothris schenckii” (#1) clustering emphasizes the importance of different strains in sporothiosis research; “american tegumentary leishmaniasis” (#4) clustering may suggest a comparison or association between sporothiosis and American skin leishmaniasis; the “golden hamster” (#9) clustering may relate to experimental investigations employing less commonly used animal models, including golden hamsters; and the “skin lesions” (#10) clustering clearly points to the sporotrichosis of skin lesion studies. These clusters demonstrate the diversity and complexity of the field of sporotrichosis research. For example, the large number of nodes in the “*sporothrix* brasiliensis” and “sporothris schenckii” clusters suggests that research on different strains has been widely followed and cited. The presence of the “action mechanism” cluster reflects the need for in-depth exploration of the pathogenesis of sporotrichosis. The importance of the “skin lesions” cluster reflects the critical position of skin lesions as the main clinical manifestation of sporotrichosis in diagnosis and treatment. In addition, the linkages between the individual clusters are of interest. The co-citation relationships between different clusters may suggest potential associations between different research directions. For example, there may be an intrinsic connection between the strain research clusters and the mechanism of action clusters and the skin lesion clusters, which together constitute the overall framework of sporotrichosis research.

**Table 3** shows two statistics, GCS (global citation score), which is the total number of citations in Web of Science, and LCS (local citation score), which is the number of citations in the current dataset. Assuming that a document exhibits a high GCS count but a low LCS, it may indicate that the document has a significant impact across various disciplines. In the context of research on *sporotrichosis*, several publications have garnered substantial attention within the academic community. For example, “The frequently cited publication entitled “*Sporothrix* brasiliensis, S. globosa, and S. mexicana: three new *Sporothrix* species of clinical interest” reflects historical taxonomic developments at the time of publication. It is important to note that subsequent phylogenetic revisions have refined species classification within the *Sporothrix* clinical clade” was published in J. Clin. Microbiol. with a high citation count (Citation of 384, GCS of 354). This suggests that the delineation of newly recognized species within the *Sporothrix* clinical clade has been of significant importance in the field and has contributed to improved taxonomic clarity and species-level diagnosis. Clinical practice guidelines for the management of sporotrichosis: 2007 update by the Infectious Diseases Society of America” was also published in the high-impact journal Clin. Infect. Dis. with a high number of citations (341 for Citation and 305 for GCS) (Kauffman et al., 2007). This suggests that the 2007 Infectious Diseases Society of America (IDSA) recommendations remain a widely cited reference in the clinical management of sporotrichosis, although no recent formal international guideline updates have been issued. “Molecular phylogeny of *sporothrix* schenckii” and “Phylogeography and evolutionary patterns in *sporothrix* spanning more than 14–000 human and animal case reports”, which focus on the molecular phylogeny and geographic distribution of *sporothrix*, also received high citation counts, reflecting the important role of basic research on sporotrichosis in advancing the field. In addition, literature on treatments for sporotrichosis, epidemiology, and studies on diseases caused by different strains of the fungus have also received a certain degree of attention. For example, “Itraconazole therapy in lymphangitic and cutaneous sporotrichosis” “Epidemiology of sporotrichosis. a study of 304 cases in Brazil” and “Feline sporotrichosis due to *sporothrix* brasiliensis: an emerging animal infection in Sao-Paulo, Brazil”, among others, have high citation counts, indicating the importance of these studies in understanding the pathogenesis and transmission of sporotrichosis and in developing effective control strategies. Overall, through the econometric analysis of the literature in the field of sporotrichosis, it can be seen that the research hotspots in this field are mainly focused on the discovery of new strains, clinical treatment guidelines, molecular phylogeny, epidemiology, and animal infections. These research results provide an important reference and basis for further in-depth study of sporotrichosis. In the future, research on these hotspots should continue to be strengthened to improve the understanding and control of sporotrichosis.

**Table 3:** Historical literature citation network in the field of sporotrichosis.

Ranking	Title	Journal	Citation	LCS	GCS	DOI	Year
1	Sporothrix brasiliensis, s-globosa, and s-mexicana, three new sporothrix species of clinical interest	J. Clin. Microbiol.	384	239	354	10.1128/JCM.00808-07	2007
2	Clinical practice guidelines for the management of sporotrichosis: 2007 update by the infectious diseases society of america	Clin. Infect. Dis.	341	156	305	10.1086/522765	2007
3	Molecular phylogeny of sporothrix schenckii	J. Clin. Microbiol.	181	105	167	10.1128/JCM.00081-06	2006
4	The divorce of sporothrix and ophiostoma: solution to a problematic relationship	Stud. Mycol.	162	62	157	10.1016/j.simyco.2016.07.001	2016
5	Phylogeography and evolutionary patterns in sporothrix spanning more than 14 000 human and animal case reports	Persoonia	163	96	150	10.3767/003158515X687416	2015
6	Sporotrichosis	Clin. Dermatol.	124	87	146	10.1086/520190	1999
7	Itraconazole therapy in lymphangitic and cutaneous sporotrichosis	Arch. Dermatol.	147	71	144	10.1001/archderm.122.4.413	1986
8	Practice guidelines for the management of patients with sporotrichosis	Clin. Infect. Dis.	141	59	132	10.1086/313751	2000
9	Sporotrichosis in peru: description of an area of hyperendemicity	Clin. Infect. Dis.	133	76	125	10.1086/313607	2000
10	Isolation and characterization of sporothrix-schenckii from clinical and environmental sources associated with the largest united-states epidemic of sporotrichosis	J. Clin. Microbiol.	127	83	122	10.1128/JCM.29.6.1106-1113.1991	1991
11	Emergence of pathogenicity in the sporothrix schenckii complex	Med. Mycol.	129	87	122	10.3109/13693786.2012.719648	2013
12	Characterization of virulence profile, protein secretion and immunogenicity of different sporothrix schenckii sensu stricto isolates compared with s. globosa and s. Brasiliensis species	Virulence	124	90	118	10.4161/viru.23112	2013
13	sporothrix species causing outbreaks in animals and humans driven by animal-animal transmission	PLoS Pathog.	117	87	114	10.1371/journal.ppat.1005638	2016
14	Epidemiology of sporotrichosis: a study of 304 cases in brazil	J. Am. Acad. Dermatol.	120	67	112	10.1016/j.jaad.2004.11.046	2005
15	Treatment of sporotrichosis with itraconazole	Am. J. Med.	112	62	110	10.1016/0002-9343(93)90280-3	1993
16	Sporotrichosis	Clin. Dermatol.	124	59	110	10.1016/j.clindermatol.2006.05.006	2007
17	Sporothrix luriei:: a rare fungus from clinical origin	Med. Mycol.	116	80	109	10.1080/13693780801992837	2008
18	Genetic diversity and antifungal susceptibility profiles in causative agents of sporotrichosis	BMC Infect. Dis.	111	78	109	10.1186/1471-2334-14-219	2014
19	Feline sporotrichosis due to sporothrix brasiliensis: an emerging animal infection in sao paulo, brazil	BMC Vet. Res.	112	77	106	10.1186/s12917-014-0269-5	2014
20	Global its diversity in the sporothrix schenckii complex	Fungal Divers.	94	62	88	10.1007/s13225-013-0220-2	2014

### 3.6 Thematic modeling

**Figure 7:**
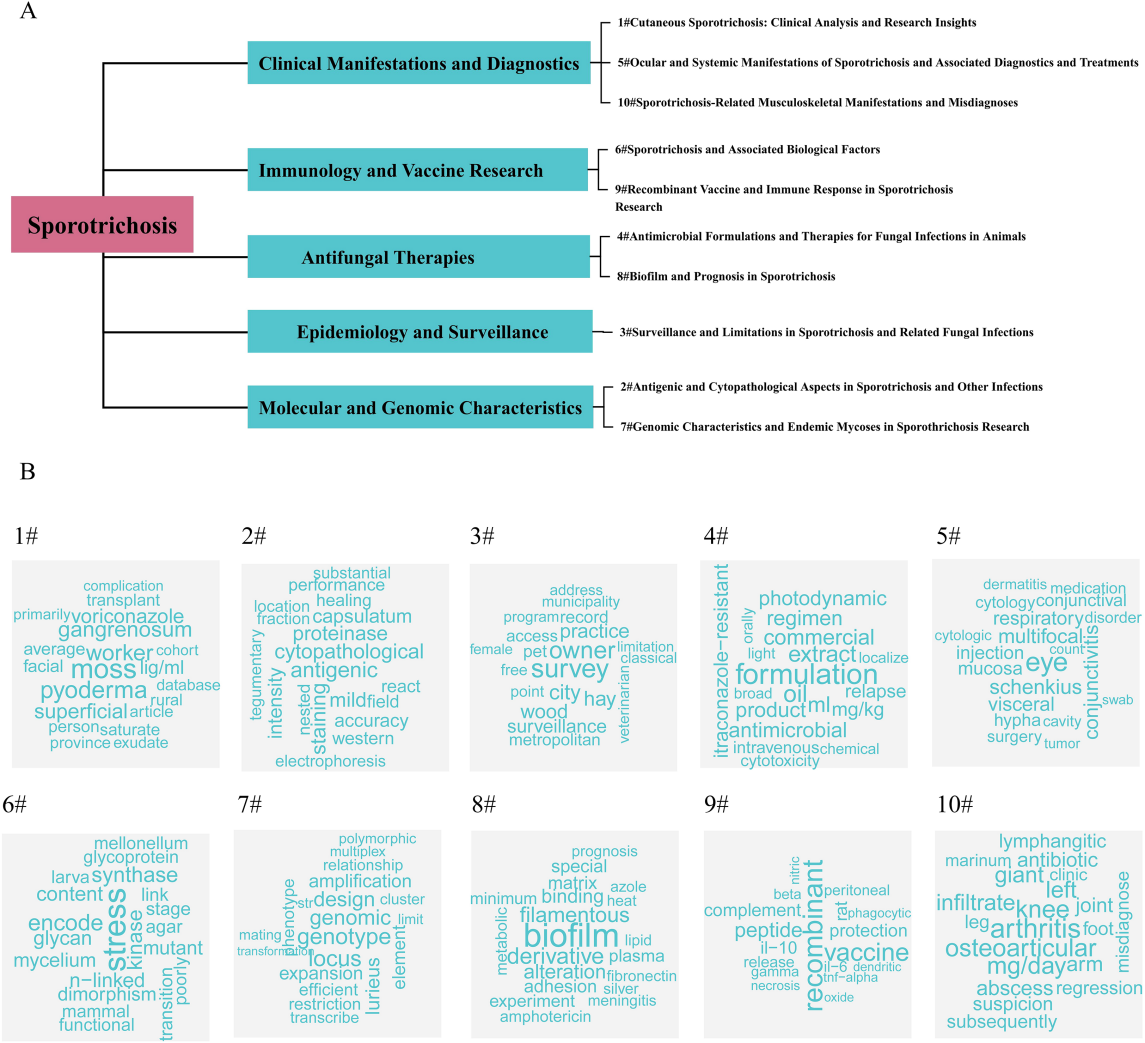
Thematic fishbone map **(A)** and keyword word cloud map **(B)** based on theme modeling.

In this study, the LDA algorithm was applied to 1,299 publications to identify the top ten research topics (**Figure 7**; **Supplementary Table 2**). Based on thematic content, these topics were consolidated into five major research directions. This structured classification reflects the multidimensional landscape of sporotrichosis research and highlights its evolving developmental trajectory (**Figure 7A**):

#### 3.6.1 Clinical manifestations and diagnostics

Sporotrichosis presents with diverse clinical forms, ranging from localized cutaneous lesions to disseminated systemic disease. Long-term cohort studies from hyperendemic regions have documented frequent extracutaneous involvement, particularly among immunocompromised patients (Fichman et al., 2022). Overlapping clinical features and yeast-like morphology pose diagnostic challenges, emphasizing the necessity of fungal isolation and molecular identification for accurate species-level confirmation in outbreak settings (Yingchanakiat et al., 2023). These findings underscore the importance of integrating clinical evaluation with laboratory confirmation.

#### 3.6.2 Immunology and vaccine research

Immunological mechanisms represent a rapidly expanding research domain. Recent studies have demonstrated that silencing of key adhesins such as GP70 and Pap1 alters cytokine responses, phagocytosis, and virulence, highlighting species-specific differences in immune modulation (Padró-Villegas et al., 2025a, 2025b). In parallel, experimental vaccine research has shown that transient depletion of regulatory T cells enhances Th1 responses and reduces fungal burden in murine models (Batista-Duharte et al., 2023). These findings indicate that host–pathogen immune interactions and antigen-based immunization strategies remain central themes in current research.

#### 3.6.3 Antifungal therapies

Antifungal treatment remains a central research focus in sporotrichosis. Recent studies have identified repurposed small molecules such as azelastine and mefloquine with inhibitory effects *in vitro* and therapeutic activity in murine models (Wang et al., 2025). High-throughput screening has also revealed olorofim as a potent candidate against pathogenic *Sporothrix* species, including antibiofilm activity (Borba-Santos et al., 2022). Clinical investigations further indicate that most *S. brasiliensis* isolates remain within wild-type susceptibility ranges, and patient outcomes are largely influenced by host factors rather than antifungal resistance (Fichman et al., 2022a).

#### 3.6.4 Epidemiology and surveillance

Epidemiological research highlights the ongoing expansion of zoonotic sporotrichosis, particularly in Brazil. Genomic epidemiology studies have demonstrated multiple independent emergence events of *S. brasiliensis* followed by sustained animal-to-human transmission across Brazilian states (Dos Santos et al., 2024). Long-term cohort data from hyperendemic areas further highlight the substantial burden of severe and disseminated disease (Fichman et al., 2022b). Emerging feline outbreaks in Southeast Asia, supported by molecular typing and antifungal susceptibility monitoring, indicate geographic spread and underscore the need for strengthened surveillance systems (Yingchanakiat et al., 2023). It should be noted that transmission mechanisms, including traumatic inoculation and zoonotic spread, did not emerge as an independent LDA topic in **Figure 7**. However, further inspection of topic-term distributions indicates that these elements were primarily incorporated within the “Epidemiology and Surveillance” cluster, where outbreak dynamics, animal-to-human transmission, and geographic dissemination were statistically grouped together. As LDA identifies themes based on probabilistic word co-occurrence patterns rather than predefined clinical classifications, conceptually central topics such as transmission may not appear as isolated categories if their associated terminology overlaps substantially with broader epidemiological constructs. Therefore, the absence of a standalone “transmission” topic reflects model-based thematic integration rather than limitations of the search strategy.

#### 3.6.5 Molecular and genomic characteristics

Molecular studies have provided important insights into the genetic diversity and evolutionary dynamics of *Sporothrix* species. Whole-genome sequencing analyses have revealed substantial single-nucleotide polymorphism differences among S. brasiliensis clades, supporting multiple independent emergence events and regional spread (Dos Santos et al., 2024). Complementary multilocus sequencing approaches using ITS, calmodulin, and β-tubulin genes have further delineated species distribution and phylogenetic clustering in outbreak settings (Yingchanakiat et al., 2023). These advances provide insight into transmission dynamics and evolutionary trajectories. Classical mycological investigations focusing on fungal morphology, dimorphism, and cell wall composition were also captured within the bibliographic dataset. In the LDA-derived thematic structure, morphology-related terms (e.g., glycoprotein, glycan, mycelium, dimorphism) were predominantly grouped within the “Molecular and Genomic Characteristics” cluster. As LDA identifies statistically coherent word distributions rather than predefined biological categories, morphology did not appear as an independent thematic title but was integrated into broader molecular-level research domains. This reflects thematic consolidation within the modeling framework rather than exclusion from the study design.

In summary, research on sporotrichosis has made remarkable progress in the areas of clinical manifestations, immune mechanisms, antifungal therapy, epidemiology and molecular characterization. Together, these interconnected research directions reflect the complexity of sporotrichosis and delineate priorities for future prevention, diagnosis, and treatment strategies.

Further analysis indicates that highly cited investigators exhibit thematic specialization aligned with the LDA-derived research domains. For instance, Anderson Messias Rodrigues and Zoilo Pires de Camargo have predominantly contributed to molecular phylogeny, species delimitation, and genomic characterization of *Sporothrix*, corresponding closely with the “Molecular and Genomic Characteristics” cluster. Almeida-Paes and Schubach have extensively investigated zoonotic transmission, clinical manifestations, and outbreak dynamics, aligning with the “Epidemiology and Surveillance” and “Clinical Manifestations and Diagnostics” domains. Similarly, Batista-Duharte and colleagues have focused on vaccine development and host immune responses, contributing to the “Immunology and Vaccine Research” cluster. These patterns suggest that influential authors are not uniformly distributed across thematic areas but tend to act as domain-specific drivers, reinforcing the structural organization revealed by topic modeling. This author–theme correspondence further validates the coherence of the LDA classification and highlights how key investigators shape active and emerging research trajectories within the field.

## 4 Bibliometric implications and future research directions

This study comprehensively presents the research status of the field of sporotrichosis through bibliometric analysis of the literature from 1975 to 2024, including research trends, disciplinary distribution, major contributing countries and institutions, key authors, and research hotspots. Nevertheless, it remains possible that a limited number of studies focusing exclusively on the genus *Sporothrix* without explicit disease reference were not captured in the present dataset. From a bibliometric perspective, the analysis identifies several research domains that remain comparatively underrepresented or methodologically fragmented within the indexed literature. While substantial progress has been made in characterizing the pathogenicity of *Sporothrix* species, the host–pathogen interaction network, particularly in immunocompromised populations, continues to represent an area of active scholarly exploration. Rather than indicating an absence of research, these patterns reflect evolving investigative emphasis across different periods and thematic clusters. The present observations are therefore intended to highlight areas of sustained academic attention and potential future expansion within the scientific literature, rather than to propose normative clinical or policy recommendations.

In terms of diagnostic approaches, current methodologies, such as histopathological examination and fungal culture, are hindered by significant limitations, including prolonged processing times and reduced sensitivity. These shortcomings impede the timely and accurate diagnosis of sporotrichosis, particularly in its early stages, where swift intervention is essential for improving clinical outcomes. There is an urgent need for the development of rapid and reliable diagnostic tools to address these clinical exigencies effectively.

The treatment landscape for sporotrichosis is equally characterized by significant challenges. The increasing prevalence of antifungal drug resistance represents a significant threat to effective management strategies. Existing treatment regimens often exhibit suboptimal efficacy in certain patient populations, resulting in inadequate clinical responses. Moreover, the prolonged administration of antifungal agents can lead to severe adverse reactions, complicating treatment protocols. Addressing these challenges necessitates ongoing research focused on novel therapeutic modalities and the identification of agents capable of overcoming resistance mechanisms while minimizing associated side effects.

Based on the above challenges, it is recommended that future research be directed towards the following specific areas:

### 4.1 Molecular characteristic research

It is recommended to further study the genome and proteome of *Sporothrix*, explore key genes and proteins related to pathogenicity and drug resistance, and provide a molecular basis for precise diagnosis and targeted therapy. For example, use gene - editing technology to study the functions of key genes, develop diagnostic methods based on molecular markers, and develop targeted drugs for specific molecular targets.

### 4.2 Immune mechanism research

It is recommended to conduct a comprehensive investigation into the host’s immune response to *Sporothrix*. This research should aim to elucidate the mechanisms of action of key immune cells and molecules involved in the anti-infection process, thereby establishing a theoretical foundation for developing immunomodulatory treatment strategies. For example, examining the synergistic interactions between cellular and humoral immunity in the context of *Sporothrix* infection, as well as the mechanisms underlying immune evasion, could reveal new targets for immunotherapy. Such studies have the potential to significantly advance our understanding and management of sporotrichosis.

### 4.3 Methods development

The advancement of sporotrichosis research necessitates the development of innovative methodologies across various domains. There is a critical need for improved diagnostic techniques that enhance sensitivity and specificity, allowing for earlier and more accurate detection of the disease. Developing rapid diagnostic tests that can be deployed in resource-limited settings would significantly impact patient outcomes and facilitate timely intervention. In addition to diagnostics, the development of novel therapeutic methods is essential. This includes the formulation of new antifungal agents with enhanced efficacy and reduced toxicity, as well as the optimization of existing treatment regimens through innovative combination therapies. Research into alternative delivery systems, such as targeted drug delivery mechanisms, can also improve treatment effectiveness while minimizing side effects.

### 4.4 Antifungal treatment research

Antifungal treatment research should prioritize two key avenues. First, there is a need to develop novel antifungal agents by exploring new drug targets, enhancing drug efficacy, and mitigating the emergence of drug resistance. This could involve designing specific inhibitors that target the unique cellular structures or metabolic pathways of *Sporothrix*. Second, optimizing combination treatment regimens of existing antifungal drugs is essential. Combining agents with different mechanisms of action can enhance therapeutic efficacy, reduce the required dosage, and minimize adverse reactions. This dual approach has the potential to improve treatment outcomes for patients with sporotrichosis and address the growing challenge of antifungal resistance.

### 4.5 Epidemiological research

Epidemiological research should emphasize the enhancement of global surveillance systems to establish a more comprehensive monitoring framework. This involves the systematic collection of detailed epidemiological data to accurately delineate the epidemic characteristics, risk factors, and transmission dynamics of sporotrichosis. Such efforts are essential for formulating targeted preventive measures and for predicting the disease’s epidemic trends. In addition, timely identification of areas prone to outbreaks will facilitate early intervention and effective control strategies, ultimately reducing the impact of the disease on affected populations. Furthermore, methodological advancements in epidemiological modeling and data analysis are crucial for understanding the dynamics of sporotrichosis transmission. Using advanced statistical techniques and computational tools can help researchers identify risk factors and predict outbreak patterns more accurately.

### 4.6 Zoonotic surveillance

Given the zoonotic nature of sporotrichosis, effective surveillance of animal populations is essential for understanding and controlling the disease’s transmission dynamics. Zoonotic surveillance should focus on identifying and monitoring potential reservoirs of *Sporothrix*, particularly in domestic and wild animals that may serve as sources of infection for humans. This involves systematic screening of at-risk animal populations, including pets, livestock, and wildlife, to detect the presence of the pathogen and assess the prevalence of sporotrichosis. Implementing robust surveillance programs can aid in identifying geographic areas with higher incidences of zoonotic transmission, thereby informing public health strategies and enabling timely interventions. Additionally, integrating veterinary and public health data can enhance our understanding of the epidemiological links between animal and human cases, facilitating a One Health approach to disease management.

### 4.7 Multidisciplinary integration

Recognizing the multidisciplinary nature of sporotrichosis research, it is essential to foster deeper integration among fields such as mathematics, computer science, materials science, and medicine. Using mathematical modeling and computer simulation technologies can enhance our ability to predict disease spread trends and assess the effectiveness of prevention and control measures. By embracing this multidisciplinary approach, we can enhance our understanding and management of sporotrichosis, leading to improved outcomes for affected populations.

### 4.8 Support for research in developing countries

Despite the contributions of developing countries to sporotrichosis research, significant disparities remain compared to their developed counterparts. To address this gap, it is imperative to enhance research support for developing nations by providing scientific funding, technical assistance, and training programs for researchers. Such initiatives would promote a more equitable distribution of global research resources. Moreover, developing countries present valuable case resources that can be leveraged to conduct large-scale clinical studies. These studies have the potential to yield critical data and insights that would enrich global sporotrichosis research. Investing in and supporting research efforts in these regions will foster innovation and collaboration, ultimately advancing our understanding and management of sporotrichosis on a global scale.

## 5 Conclusion

This bibliometric analysis of sporotrichosis research highlights its interdisciplinary nature and the increasing recognition of the disease as a significant public health concern. The findings indicate a substantial rise in publications and citations, particularly from Brazil, reflecting the disease’s prevalence in the region.

Key insights reveal influential authors, institutions, and journals shaping the field, along with five major research directions identified through LDA: clinical manifestations, epidemiology, antifungal therapies, immunological responses, and molecular characteristics. Bibliometric trends indicate sustained scholarly attention to diagnostics and antifungal resistance, with increasing publication activity related to therapeutic innovation and diagnostic refinement; however, the present analysis does not directly evaluate clinical efficacy, translational applicability, or guideline-level validation.

The study emphasizes the critical need for international collaboration and resource sharing to address these challenges. Enhanced support for research in developing countries is essential for advancing knowledge and improving public health outcomes. Prioritizing interdisciplinary approaches allows the research community to effectively address the complexities of sporotrichosis, resulting in improved prevention and treatment strategies. Overall, this analysis provides the basis for future investigations, guiding efforts to fill critical gaps in understanding this impactful infectious disease.

## Data Availability

The original contributions presented in the study are included in the article/**Supplementary Material**. Further inquiries can be directed to the corresponding authors.

## Supplementary material

Supplementary Figure 1 Theme number selection curve.

Supplementary Table 1 Corpus data.

Supplementary Table 2 Thematic naming materials.
