# Supplementary figures and images for: Combining disciplines in the field of modern microbiology: a quantitative analysis of the literature on sporotrichosis
Source: Front Cell Infect Microbiol. 2026 Apr 20;16:1742934. doi: 10.3389/fcimb.2026.1742934 (PMC13135933; doi:10.3389/fcimb.2026.1742934)

minimize

metrics:

- Griffiths2004
- ▲ CaoJuan2009
- Arun2010
- + Deveaud2014

maximize

2 3 4 5 6 7 8 9 10 11 12 13 14 15 16 17 18 19 20 21 22 23 24 25 26 27 28 29 30 31 32 33 34 35 36 37 38 39 40 41 42 43 44 45 46 47 48 49 50

number of topics

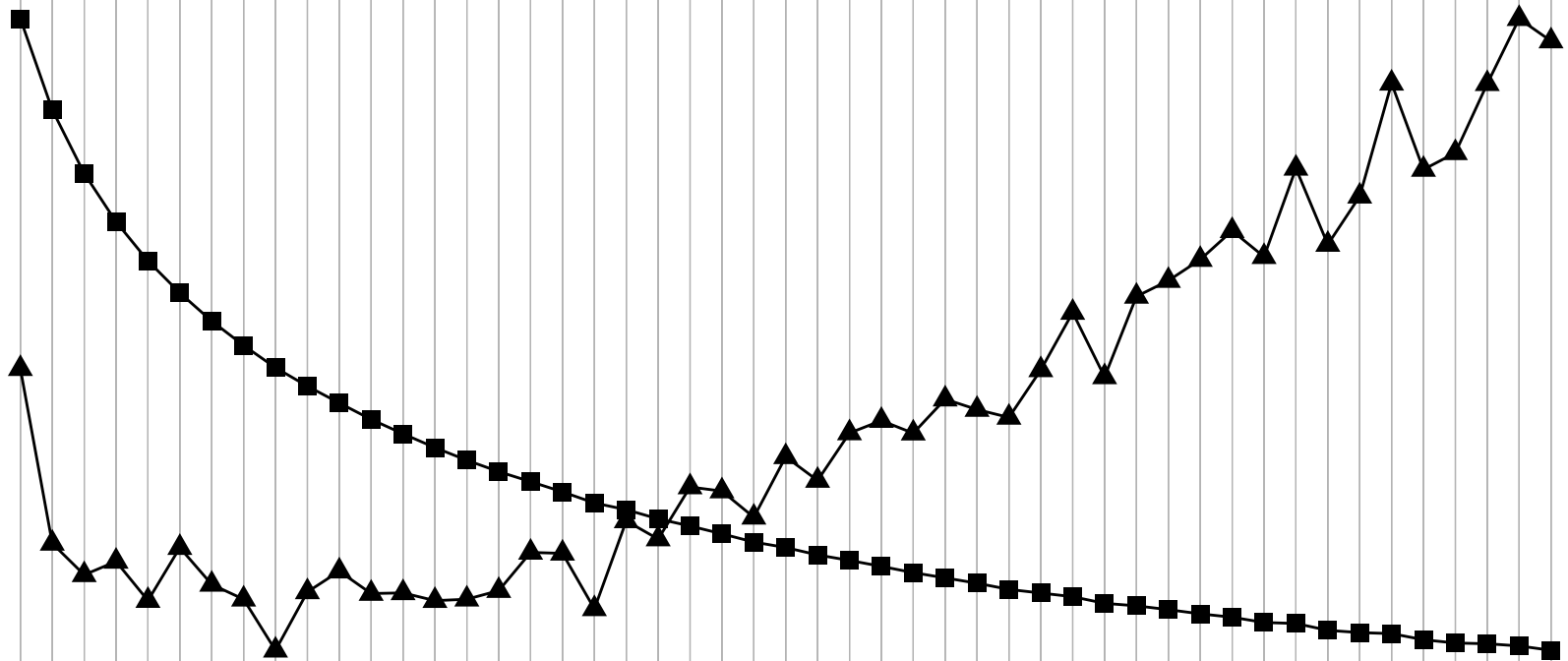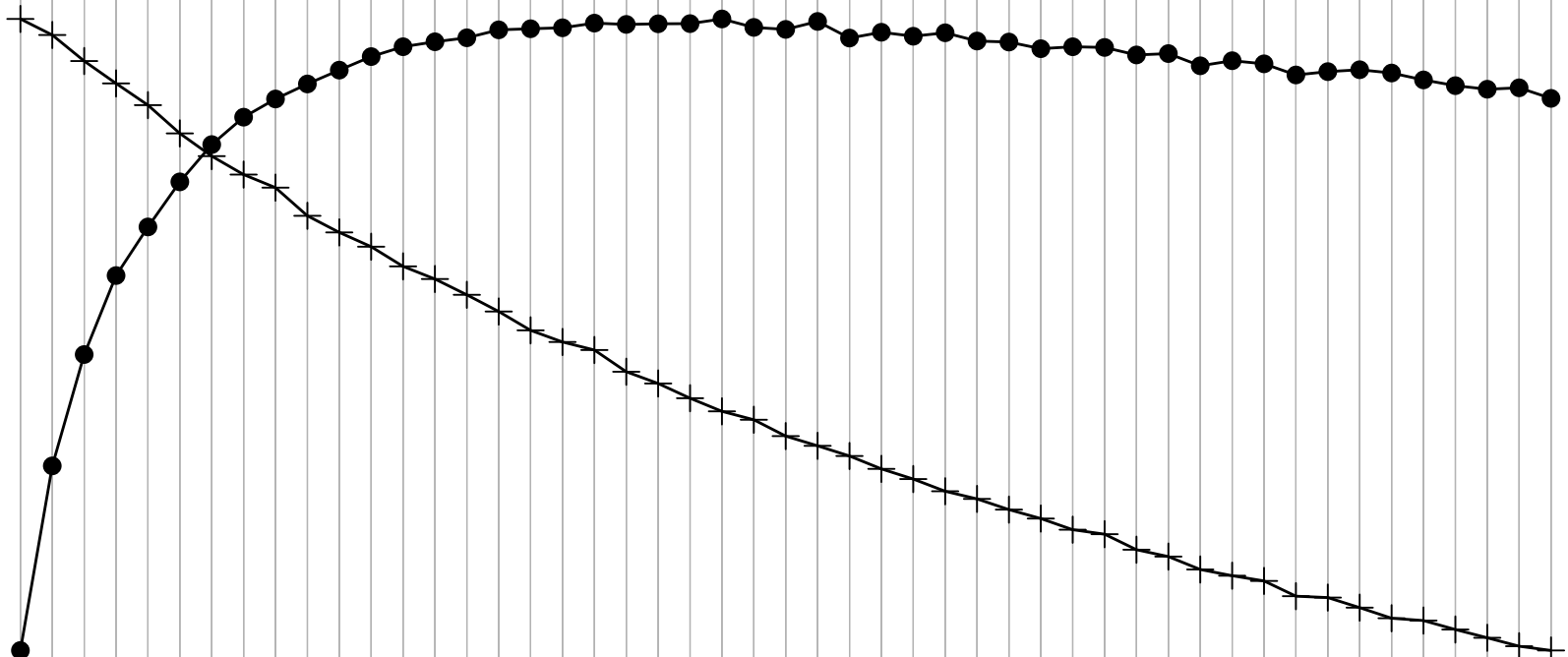

Supplement: Supplementary Figure 1 — Theme number selection curve. [file Image1.pdf]
